# A preliminary study on designing a cluster randomized control trial of two new mosquito nets to prevent malaria parasite infection

**DOI:** 10.1186/s41182-020-00276-x

**Published:** 2020-12-07

**Authors:** Noboru Minakawa, James O. Kongere, George O. Sonye, Peter A. Lutiali, Beatrice Awuor, Hitoshi Kawada, Rie Isozumi, Kyoko Futami

**Affiliations:** 1 Institute of Tropical Medicine (NEKKEN), Nagasaki University, 1-12-4 Sakamoto, Nagasaki, 852-8523 Japan; 2 Kenya Medical Research Institute, Nairobi, Kenya; 3 Center for Research in Tropical Medicine and Community Development (CRTMCD), Nairobi, Kenya; 4 Ability to Solve by Knowledge Project, Mbita, Kenya

**Keywords:** Malaria, LLIN, PBO, Ceiling, Study design, RCT

## Abstract

**Background:**

Although long-lasting insecticidal nets (LLINs) are the most effective tool for preventing malaria parasite transmission, the nets have some limitations. For example, the increase of LLIN use has induced the rapid expansion of mosquito insecticide resistance. More than two persons often share one net, which increases the infection risk. To overcome these problems, two new mosquito nets were developed, one incorporating piperonyl butoxide and another covering ceilings and open eaves. We designed a cluster randomized controlled trial (cRCT) to evaluate these nets based on the information provided in the present preliminary study.

**Results:**

Nearly 75% of the anopheline population in the study area in western Kenya was *Anopheles gambiae s. l.*, and the remaining was *Anopheles funestus s. l*. More female anophelines were recorded in the western part of the study area. The number of anophelines increased with rainfall. We planned to have 80% power to detect a 50% reduction in female anophelines between the control group and each intervention group. The between-cluster coefficient of variance was 0.192. As the number of clusters was limited to 4 due to the size of the study area, the estimated cluster size was 7 spray catches with an alpha of 0.05. Of 1619 children tested, 626 (48%) were *Plasmodium falciparum* positive using a rapid diagnostic test (RDT). The prevalence was higher in the northwestern part of the study area. The number of children who slept under bed nets was 929 (71%). The *P. falciparum* RDT-positive prevalence (RDTpfPR) of net users was 45%, and that of non-users was 55% (OR 0.73; 95% CI 0.56, 0.95). Using 45% RDTpfPR of net users, we expected each intervention to reduce prevalence by 50%. The intracluster correlation coefficient was 0.053. With 80% power and an alpha of 0.05, the estimated cluster size was 116 children. Based on the distribution of children, we modified the boundaries of the clusters and established 300-m buffer zones along the boundaries to minimize a spillover effect.

**Conclusions:**

The cRCT study design is feasible. As the number of clusters is limited, we will apply a two-stage procedure with the baseline data to evaluate each intervention.

## Background

Insecticide-treated nets (ITNs) are the most effective tool for preventing malaria parasite infection [1–3]. With an increase of ITN coverage, the infection prevalence in endemic Africa halved between 2000 and 2015, and the clinical cases fell by 40% [4, 5]. Although ITNs were continuously delivered to high-risk areas, the rate of reduction slowed dramatically from 2014 to 2018 [6, 7]. Without a significant change in the current strategy, it will be difficult to reach the goals for malaria elimination set by the Global Technical Strategy for Malaria 2016–2030 [7].

According to the World Malaria Report 2019 by the World Health Organization (WHO), half of the people at risk of malaria in sub-Saharan Africa were sleeping under ITNs in 2018, and households with at least one ITN for every two people increased to 72% [7]. However, this is still far from realizing universal coverage. Children often share one net with more than two persons [8–10]. Even when the number of ITNs is sufficient to cover all family members, their sleeping spaces are limited for hanging them [11]. Under crowded conditions, the risk of infection may increase because children touch the net, and extremities extend or persons roll outside the net. In particular, when sleeping on the floor, it becomes difficult for small children to hang nets properly [10, 12].

House screening may reduce the risk for children under these conditions. A study with experimental huts in Gambia reported that screened ceilings and eaves reduced entry of *Anopheles gambiae* sensu lato (*s. l.*) by about 80% [13]. In western Kenya, a similar study confirmed the effects of screening against *An. gambiae s. l.* and *Anopheles. funestus s. l.*, and their densities remained low for 9 months, until removal of the nets [14]. The Kenya study used a fabric of long-lasting insecticidal nets (LLINs) to screen ceilings and eaves while the Gambia study used non-insecticidal nets. A randomized controlled trial in Gambia showed that the number of anopheline mosquitoes was reduced by about 50% in houses with screened ceilings [15]. The study also found that screened ceilings reduced the number of children with anemia (Hb < 8 g/dL) by 50%. However, the difference in frequency of parasitemia was not statistically significant between the control and intervention groups.

Along with indoor residual spraying (IRS), the increase of LLIN use has induced the rapid expansion of vectors resistant to pyrethroids. This has become the most serious threat to the current malaria control program because synthetic pyrethroids are mainly used for LLINs [16, 17]. To date, malaria vectors have developed two main resistance mechanisms, target site resistance and metabolic resistance [18]. The target site resistance has a point mutation at 1014L (L1014F or L1014S) within a voltage-gated sodium channel. This mutation causes insensitivity to pyrethroids, resulting in knockdown resistance (*kdr*) [19]. The metabolic resistance is related to the elevated activity of one or more detoxification enzymes (cytochrome P450s) [20, 21].

To overcome the insecticide resistance issue, LLINs incorporating piperonyl butoxide (PBO) have been developed. PBO is a synergist to inhibit the activities of the enzymes that enhance resistance of mosquitoes by metabolizing pyrethroids. Experimental hut trials showed that PBO-LLINs outperform standard LLINs without PBO against resistant malaria vectors [22–26]. A systematic review revealed that PBO-LLINs increase mosquito mortality by 84% compared with standard LLINs in areas where mosquitoes have high pyrethroid resistance [27]. The cluster randomized controlled trial (cRCT) in Tanzania reported that malaria infection prevalence was lower in the group that received PBO-LLINs than in the group that received standard LLIN after 9 months, and the effectiveness was sustained after 21 months [28]. The cRCT in Uganda also found that parasite prevalence was lower in areas covered with PBO-LLINs compared with standard LLINs [29].

We planned a cRCT to evaluate PBO-LLINs and ceiling nets made of an LLIN fabric in an area with resistant anophelines in western Kenya. As the previous ceiling net study in Gambia did not use an LLIN fabric [15], we will use the Olyset® Net fabric which is incorporated with 2% permethrin (Sumitomo Chemical, Tokyo, Japan). The PBO-LLIN studies in Tanzania and Uganda were conducted in an area where *An. gambiae sensu stricto* (*s. s.*) with a high level of *kdr* was predominant [28, 29]. In contrast, we planned a cRCT in an area where *Anopheles arabiensis* and *An. funestus s. s.* with metabolic resistance are predominant [30–32]. As PBO is more effective against metabolic resistance, the present study site is more appropriate than the Tanzania study site to test PBO-LLIN.

The objective of this preliminary research was to obtain the entomological and epidemiological background information for designing a study protocol of the cRCT to test these two new malaria control tools. The study protocol included estimating the sample sizes for the primary endpoints, specifically the abundance of female anopheline mosquitoes and *Plasmodium falciparum* positive prevalence of children under 10 years of age.

## Methods

### Study area

**Fig. 1:**
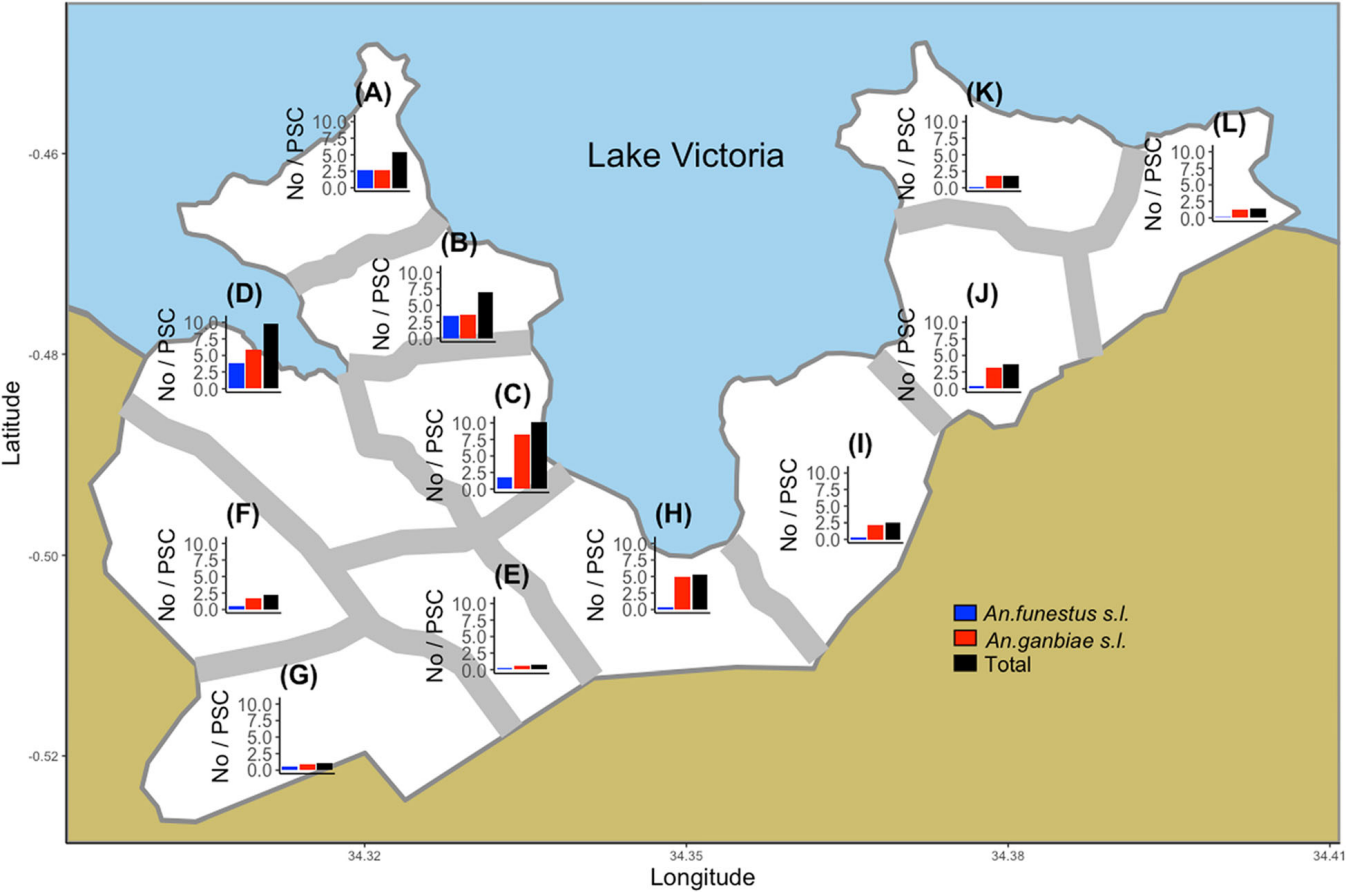
The boundaries with buffer zones between clusters and the numbers of anopheline mosquitoes. *Anopheles funestus s. l.* and *An. gambiae s. l.* were collected biweekly from 10 sentinel houses within each cluster using the pyrethrum spray catch method during the period from April 2009 to April 2010

The study area was Gembe East of Homa Bay County in western Kenya. The total land area is approximately 46 km^2^, and the coordinates of the geographical center are 0° 30′ 24″ S and 34° 20′ 48″ E. The area was divided into 12 clusters based on the boundaries of 14 villages or communities (Fig. 1). Most houses are constructed using a stick framework plastered with a mixture of mud and cow dung, and a corrugated iron roof. A past study in the area reported that the median number of rooms per house was 2, and the mean room size was 11 m^2^ [10]. Nearly 90% of houses had open eaves [33]. Most residents belong to the Luo ethnic group. Although Dholuo is the main language spoken, most residents speak English and Kiswahili. The main income sources are fishing, traditional small-scale farming, and cattle breeding [34].

### Entomological data

We obtained entomological data from a sentinel surveillance system that a past study established to monitor house-resting anopheline mosquitoes in the study area and the adjacent area [30]. Mosquitoes were collected biweekly from 10 sentinel houses within each cluster using the pyrethrum spray catch method. We selected 5 or 10 traditional houses with open eaves from the outer edge of each community center, because the community centers have few traditional houses. A total of 120 houses in the study area had been monitored for temporal changes in abundance and species composition of female anophelines. Sampled anophelines were divided to *An. gambiae s. l.* and *An. funestus s. l.* under the microscope, and their numbers were recorded.

### Epidemiological data

Prior to the epidemiological survey, we held a series of meetings with the local chiefs, assistant chiefs, opinion leaders, village elders, and the district medical officers, and explained to them the goals and purpose of this study. After consulting with the local administration, we investigated *P. falciparum* infection prevalence among children from 7 to 119 months old in July 2010. A list of target children was obtained from the demographic surveillance system [35]. We asked the local community leaders and health workers to inform caretakers of the school and community center testing locations and dates.

Axillary temperature of each child was measured, and a finger prick blood sample was taken to examine *P. falciparum* infection with a rapid diagnostic test (RDT; Paracheck-Pf, Orchard Biomedical System, Goa, India) and to measure hemoglobin concentration with a portable hemoglobin photometer (Hemocue, Angelholm, Sweden). Artemether-lumefantrine was given to each child who had a positive RDT and body temperature above 37.5 °C. However, some children whose symptoms did not follow the above criteria were also given the treatment based on WHO guidelines and diagnosis by a clinician [36]. Children with hemoglobin concentration below 11 g/dL were given iron supplementation.

While waiting for the results of the RDTs, caretakers were interviewed on whether their children slept under an LLIN the previous night, a standard protocol to assess LLIN use [37–40]. A previous study in the adjacent area found that the result from interviews for LLIN use was similar to that from direct observations in the early morning [34].

### Statistical analysis

We used a logistic regression model for revealing the relationships of *P. falciparum* RDT-positive prevalence (RDTpfPR) with age and bed net use. Spatial dependency was incorporated in the regression model using Bayesian statistics (R package: INLA) [41]. The spatial pattern of RDTpfPR was revealed using posterior mean values of the spatial field estimated with the Bayesian regression model [42]. Similarly, the spatial pattern of bed net use was analyzed with a Bayesian logistic regression model.

## Results

### Entomological data

**Fig. 2:**
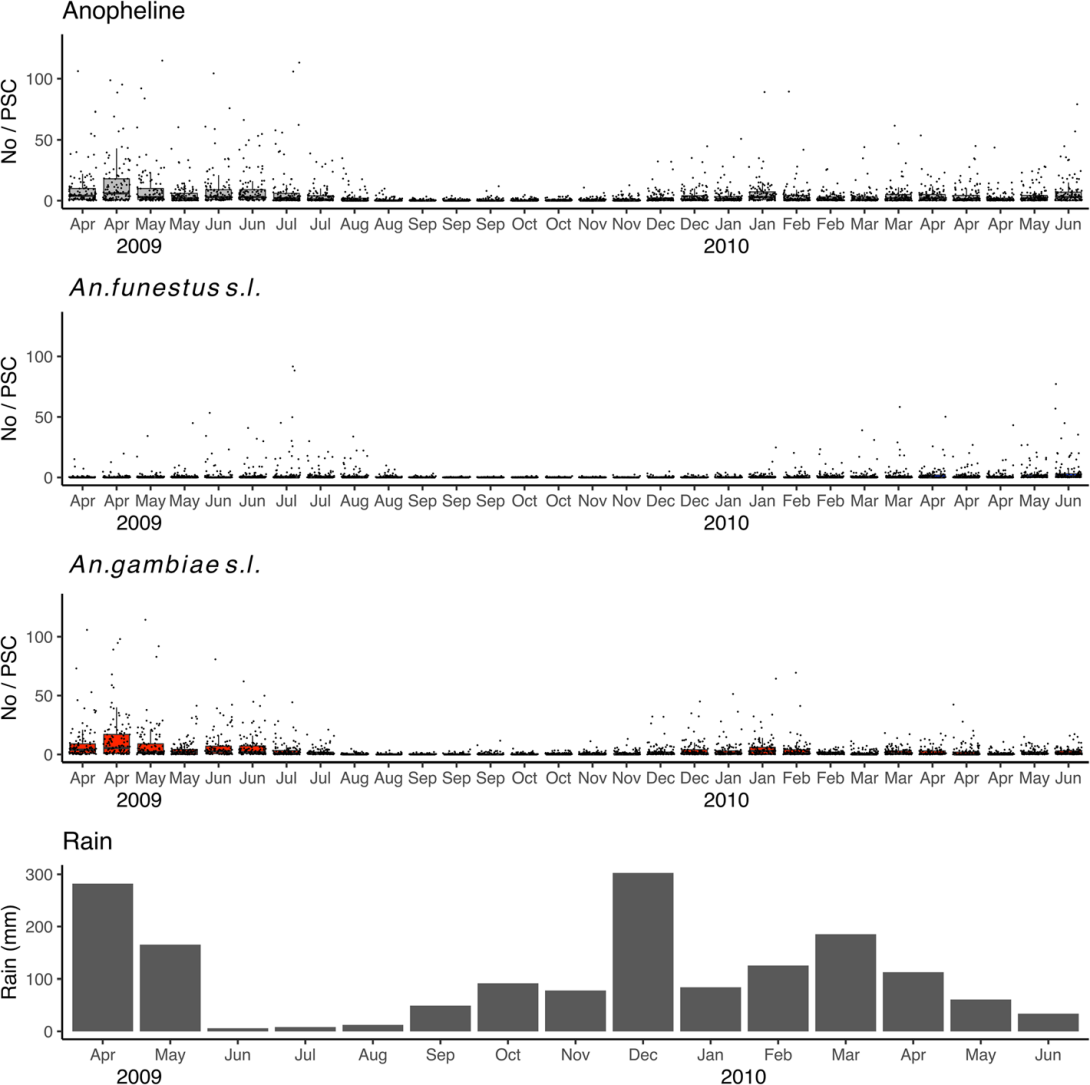
Temporal changes in the numbers of anopheline mosquitoes and monthly rainfall. *Anopheles funestus s. l.* and *An. gambiae s. l.* were collected biweekly from 10 sentinel houses within each cluster using the pyrethrum spray catch method during the period from April 2009 to April 2010. The rainfall data were obtained from an automated weather station (AWS) in the ICIPE Thomas Odhiambo Campus, Mbita, Homabay County, Kenya. The closest distance from the study area to the AWS was approximately 11 km

A total of 15,281 house-resting female anophelines were collected during the period from April 2009 to April 2010. The number of anophelines per spray catch was 4.3 (the median = 1, IQR 0–4, *n* = 3600). The numbers of *An. gambiae s. l.* and *An. funestus s. l.* were 11,155 (73%) and 4126 (27%), respectively. More anophelines were recorded in the western part of the study area (Fig. 1). The number and proportion of *An. funestus s. l.* were greater in the western area compared with the eastern part. The number of anophelines increased during the long rainy seasons of 2009 and 2010 (Fig. 2). The long rainy season typically occurs during the months of April, May, and June. The number of *An. gambiae s. l.* also increased during the period from December 2009 to February 2010 when high rainfall was recorded.

### Epidemiological data

**Fig. 3:**
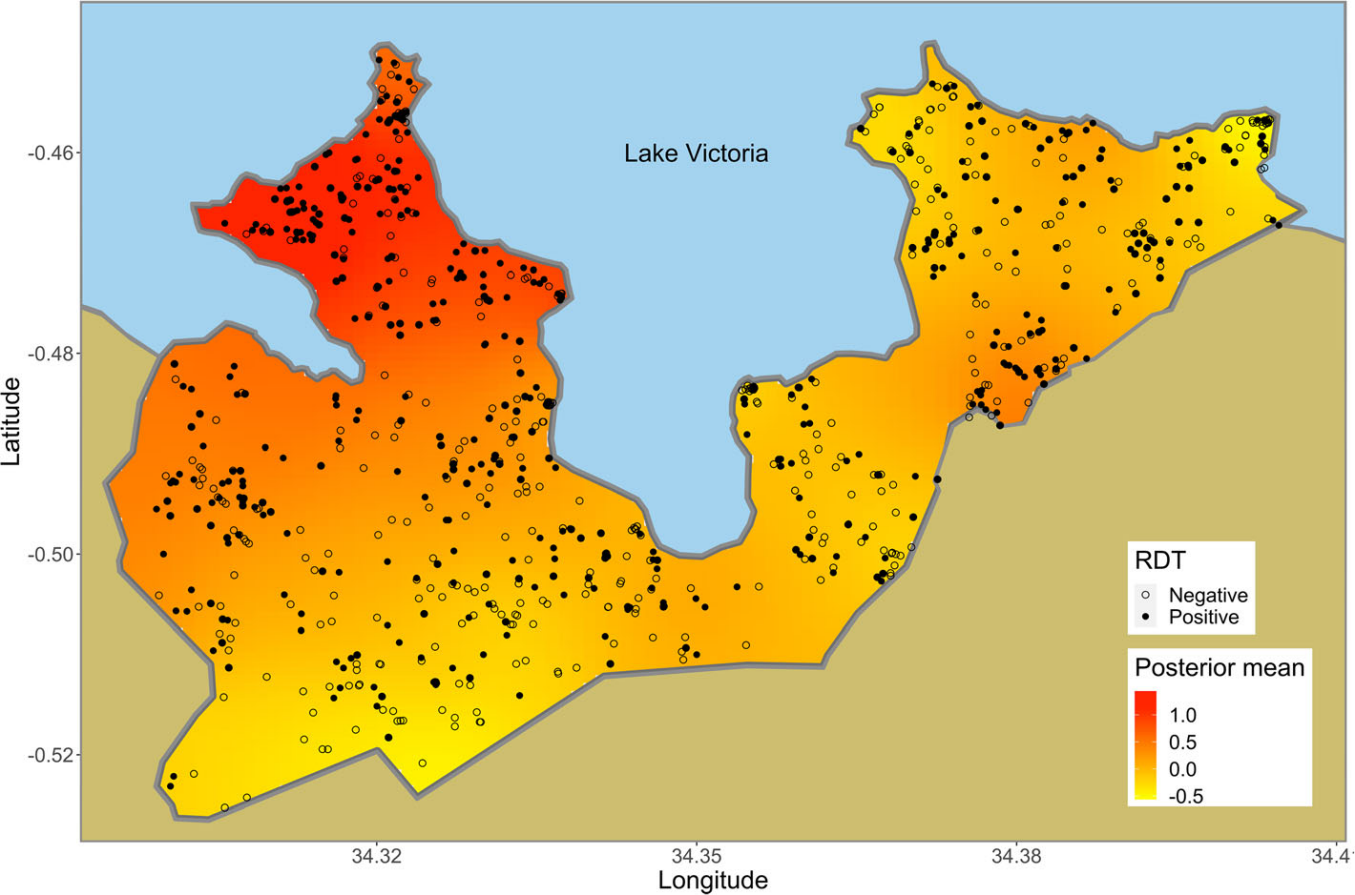
The interpolated spatial pattern of RDTpfPR. The map was generated with posterior mean values of spatial random field obtained from the Bayesian regression model

**Fig. 4:**
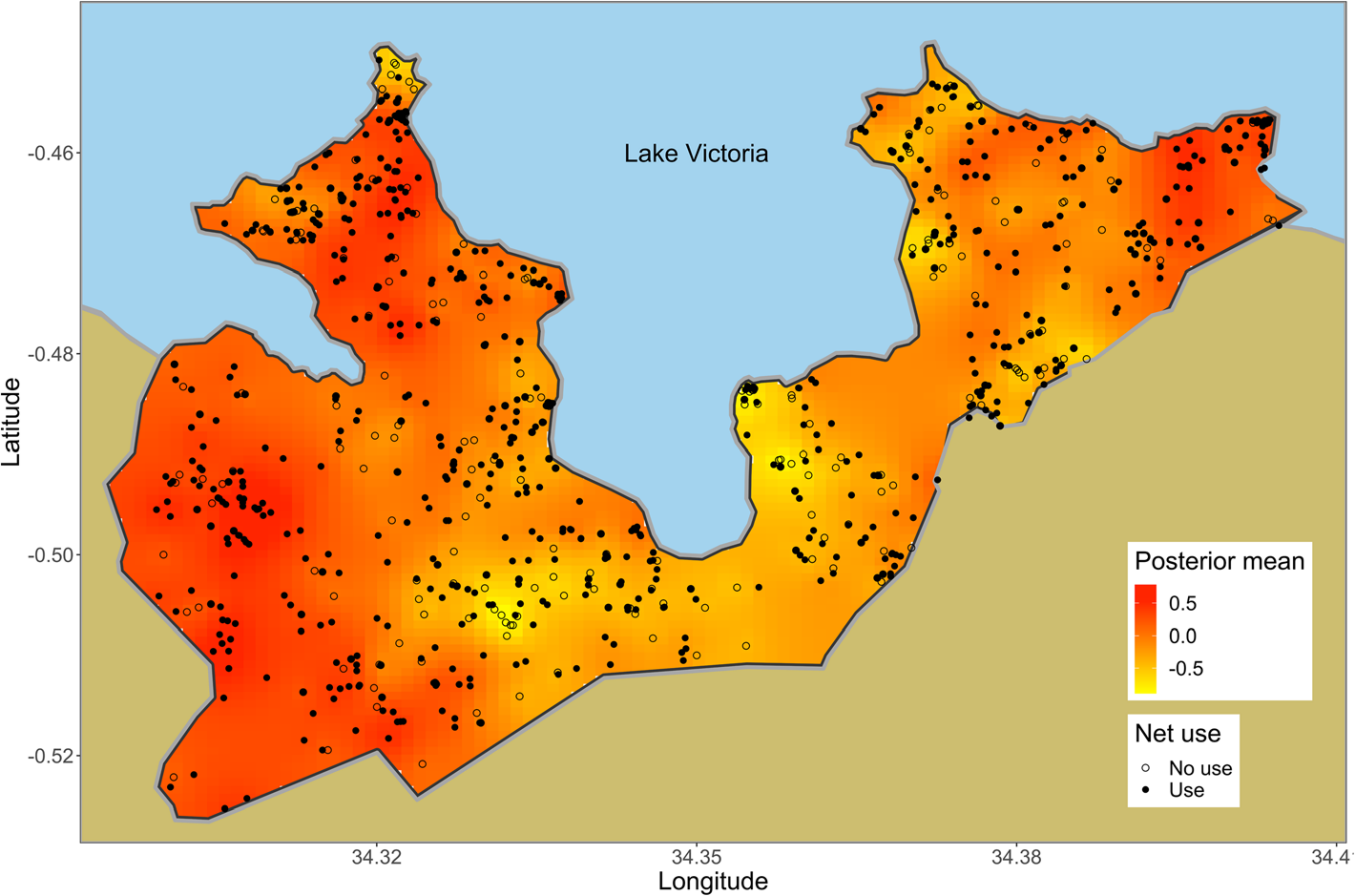
The interpolated spatial pattern of net use. The map was generated with posterior mean values of spatial random field obtained from the Bayesian regression model

**Fig. 5:**
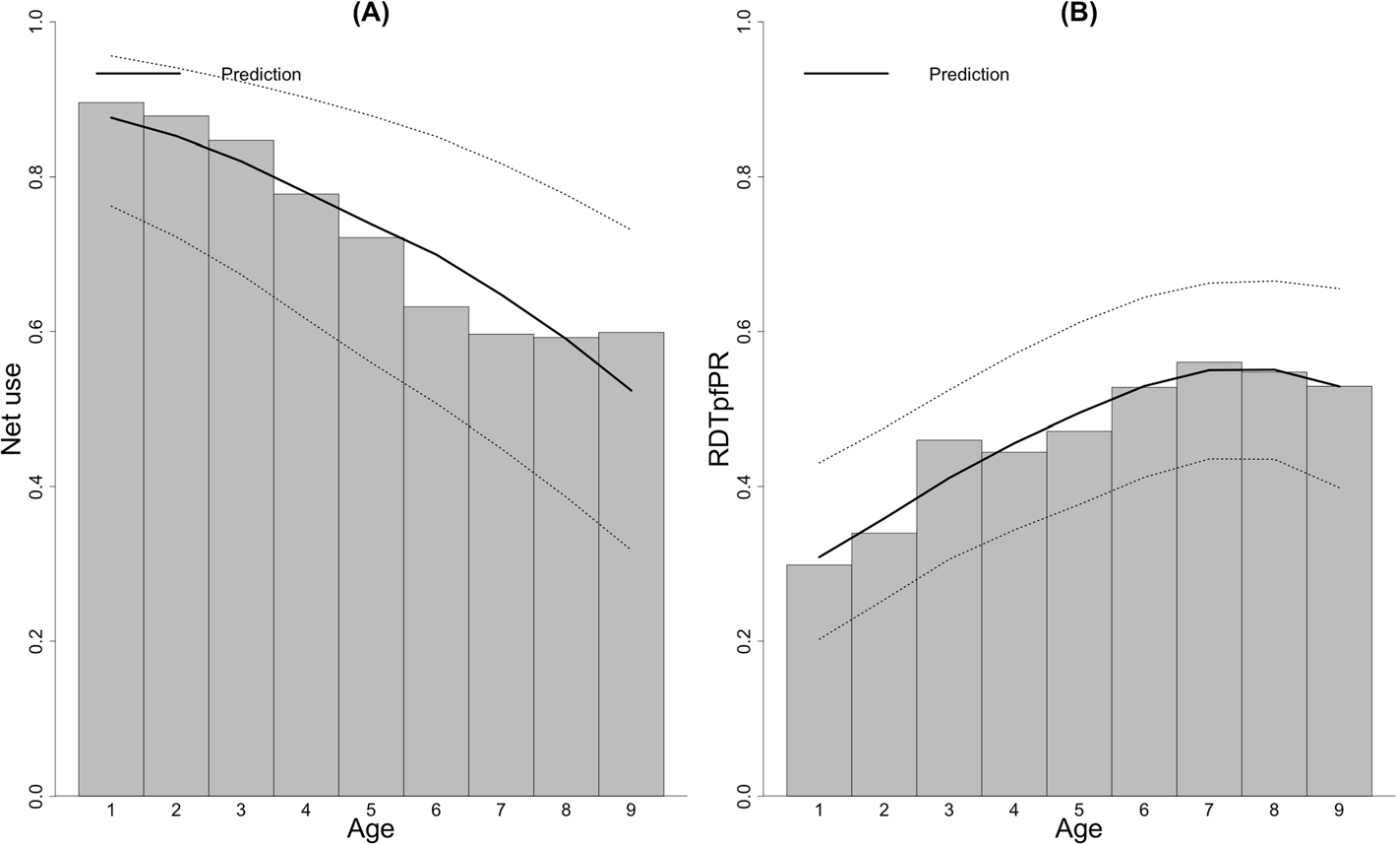
The relationships of age with RDTpfPR and net use

We could not locate 793 of 3896 children targeted. These children either had migrated to other areas or had been temporarily absent from their homes when the field assistants visited. Since caretakers of 592 children were absent from their homes, we could not obtain consent from them. Caretakers of 8 children refused participation. Of 2503 remaining children, 884 children did not appear at the testing centers. The survey tested 1619 children, which was 42% of the target population. As the dataset of 310 children lacked complete information such as age, house location, and bed net use, the dataset of 1309 remaining children was used for the analyses. Of them, 626 children (48%) tested positive with RDT. The RDTpfPR was spatially heterogeneous, and the prevalence was higher in the northwestern part of the study area (Fig. 3). The number of children who slept under bed nets was 929 (71%). The bed net use was spatially heterogeneous and was lower in the central part of the study area (Fig. 4). The bed net use decreased with an increase in age (Fig. 5a). The RDTpfPR of net users was 45%, and that of non-users was 55%. The prevalence increased up to age 7 and decreased thereafter (Fig. 5b). To predict RDTpfPR, the optimal regression model included four covariates: bed net use (OR 0.73; 95% CI 0.56, 0.95), age (OR 1.28; 95% CI 1.06, 1.15), age-squared (OR 0.88; 95% CI 0.77, 1.07), and spatial dependency.

## Study design of cRCT

### Study area

Based on the distribution of children, we modified the boundaries to keep distance from populated areas; specifically, we tried to create a “fried-egg” design [43]. The mean area of the clusters was 3.8 km^2^ (sd = 0.86). We also established 300-m buffer zones along the boundaries to minimize a spillover effect between clusters (Fig. 1) [44, 45].

### Sample size calculation

The number of female anophelines per spray catch was one of the primary endpoints. The previous study of screened ceilings in experiment huts reported an 80% reduction of house entering by *An. gambiae s. l.* [13], and the RCT in Gambia reported a 47% reduction of *An. gambiae s. l.* [15]. In an area where *An. gambiae* with high level of *kdr* was predominant, the mortality rate by PBO-LLINs was 78% while it was 44% with standard LLINs [22]. Therefore, we planned to have 80% power to detect a 50% reduction in female anophelines between the control group and each intervention group. Since the data from the present study was over-dispersed, we fitted a negative binomial regression model for the estimation. The between-cluster coefficient of variance was 0.192. As the number of clusters was limited to 4 due to the size of the study area, the estimated cluster size was 7 spray catches with an alpha of 0.05.

We decided to use *P. falciparum* positive prevalence based on polymerase chain reaction (RCR) for the epidemiological primary endpoint, because PCR has higher sensitivity and specificity compared with RDT and microscopy that were used by the previous studies [15, 28]. Using 45% RDTpfPR of net users in the present study, we also expected each intervention to reduce *P. falciparum* PCR-positive prevalence (PCRpfPR) by 50%. The estimated ICC was 0.053. With 80% power and an alpha of 0.05, the estimated cluster size was 116 children. Although RDT may produce false positives, the discrepancy is small enough to estimate the sample size for PCRpfPR using the data from RDT [46]. We had a high expectation for the effectiveness, because nearly all vectors had acquired metabolic resistance in this study area [30–32]. A synergy effect was also expected with ceiling nets and standard LLINs.

### Baseline survey

Since the number of clusters is the minimum requirement for a cRCT, the baseline data obtained before the intervention will be used for adjusting imbalances among the clusters, which will increase statistical power [43, 47]. For an entomological baseline, we will use the data from the sentinel surveillance during the period between April 2009 and February 2011.

**Fig. 6:**
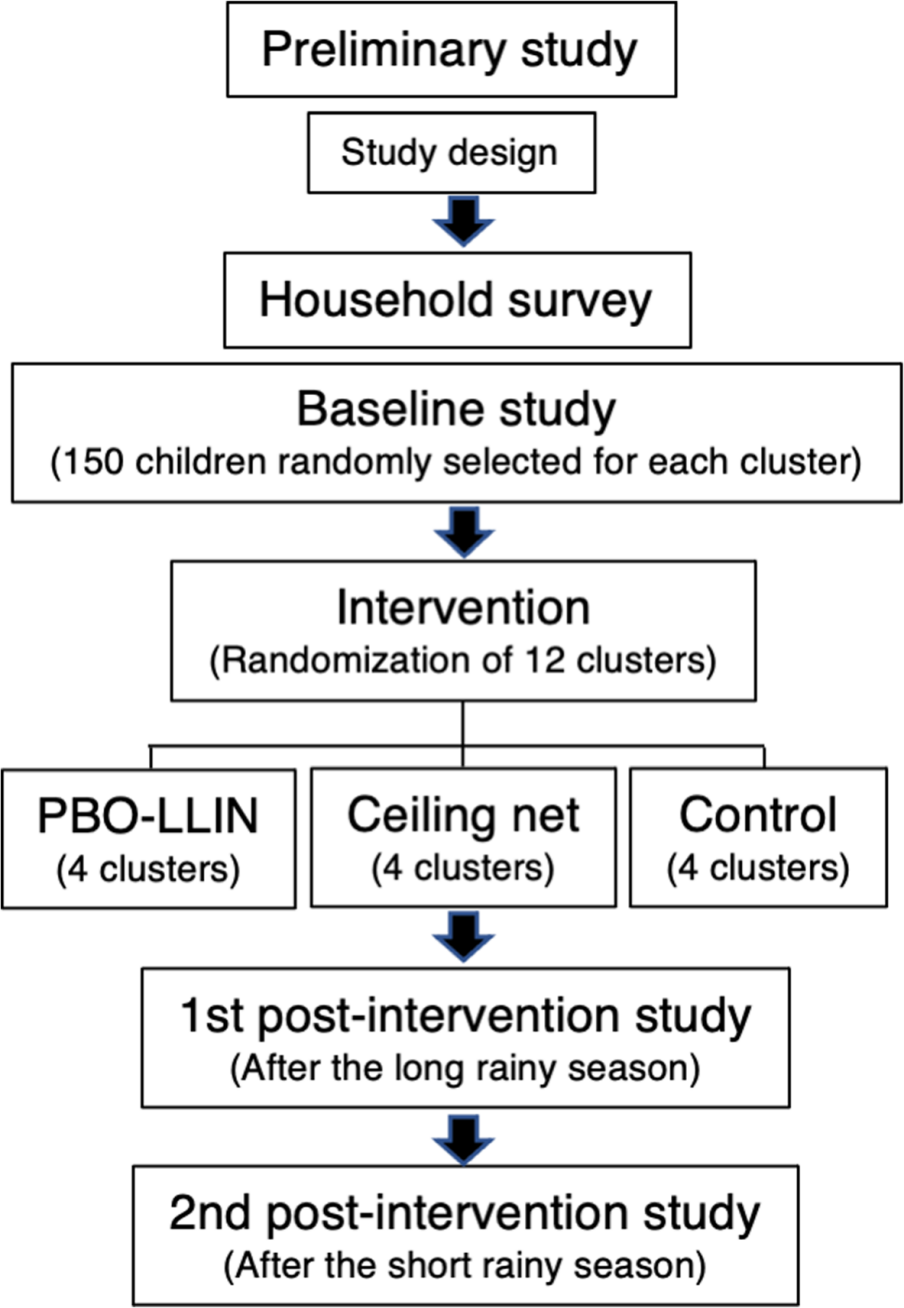
Flowchart of the study design

For an epidemiological baseline, we will update the list of children aged 7 months to 10 years old through a house survey (Fig. 6). The household survey will also record information to assess socioeconomic status (SES) and house condition. The SES for each household will be estimated using a composite household material wealth index based on possession of various consumer goods, house construction, toilet/water access, and livestock [48, 49]. A numerical score will be assigned to each household using multiple corresponding analysis. The continuous measures will be then divided into tertiles to obtain a rough proxy of SES [49]. Using the list, we will randomly select 150 children for each cluster. We will inflate the sample size because of anticipated dropouts. As the appearance rate of children at the testing sites was low in the present preliminary study, the trained field assistants will visit households of the children 1 day before the survey to remind caretakers. The testing sites will be established in easily accessed schools and community centers. The screening test will follow the same procedure as the preliminary study. Blood samples will be collected on filter papers for PCR. For mosquitoes, we will use the data from the sentinel surveillance as a baseline.

### Intervention

Immediately after the epidemiological baseline survey, we will randomly select 4 clusters for each PBO-LLIN intervention, ceiling net intervention, and control (Fig. 6). We will provide enough PBO-LLINs (Olyset® Plus, Sumitomo Chemical, Tokyo, Japan) to all houses in the PBO-LLIN intervention clusters based on the WHO recommendation (at least 1 LLIN for every 2 persons) [9]. For house with an odd number of residents, we will provide extra LLINs to ensure that all persons will have access to a bed net (e.g., 2 nets for 3 persons, and 3 nets for 5 persons). In the control clusters, we will provide enough standard LLINs (Olyset® Net, Sumitomo Chemical, Tokyo, Japan) to all houses. The two types of LLINs have the same color and shape, and similar texture. They will be distinguishable only by a unique code on the label, and residents and field assistants will not be told which will be the PBO-LLIN. The old bed nets will be removed from the houses with consent.

We will also provide enough standard LLINs to all houses in the ceiling net clusters, and install ceiling nets in all houses except concrete houses without open eaves. The Olyset® Net fabric will be cut and sewn into a sheet measuring 7 × 5 m. Depending on the size and shape of the house, 1 to 2 sheets are usually required to cover the ceiling and eaves of a single house. We will train local residents for installation of ceiling nets. The details of the ceiling net were described in the previous study in this study area [14].

### Post-intervention survey

We will use the entomological data from the sentinel surveillance after the intervention. Because the sentinel houses were not randomly selected, we will also conduct cross-sectional surveys with 25 randomly selected houses in each cluster during the long rainy season. All selected houses will have one room and open eaves. Indoor-resting female anopheline mosquitoes will be sampled using the pyrethrum spray catch method. Immediately after the short rainy season, the entomological cross-sectional survey will be repeated selecting 25 houses randomly for each cluster.

The post-intervention epidemiological survey will be conducted after the long rainy season and repeated after the short rainy season following the same procedure as the baseline survey. The list of children will be updated prior to the second post-intervention survey.

### Statistical analysis

The effects of PBO-LLINs and ceiling nets will be separately evaluated because of the small number of clusters [43, 47]. A two-stage procedure with baseline data will be applied to evaluate each intervention. In the first stage, we used a regression model to obtain a residual for each cluster that will be adjusted for the covariates. The covariates of interest will be age, age-squared, bed net use, sleeping location, SES, and baseline. In the second stage, Wilcoxon’s rank sum test will be used to compare the residuals of two arms.

## Discussion

In this preliminary study, the difference in RDTpfPR was 23% between two groups of children under 10 years of age; however, the RDTpfPR of net users was still high. The high prevalence in the study area is partially explained by a vector population that has developed insecticide resistance. *Anopheles arabiensis* and *An. funestus s. s.* in this study area have developed metabolic resistance related to one or more detoxification enzymes [31, 32]. The present study showed that mosquitoes belonging to *An. funestus s. l.* were abundant in the western part where extensive wetland occurs along the lake shore [50], and the past study showed that they are mostly *An. funestus s. s* [30]. Throughout the study area, *An. gambiae s. l.* was more abundant than *An. funestus s. l.* The mosquitoes belonging to *An. gambiae s. l.* are mostly *An. arabiensis* while *An. gambiae s. s.* which has developed *kdr* is apparently disappearing from the area [30]. As PBO inhibits the activity of detoxification enzymes, we expected a considerable impact of PBO-LLINs on the anopheline population in the present study area [51].

Nearly 90% of the houses in our study area had open eaves [33]. Eave openings are the main entrance of anopheline mosquitoes [52]. The study in Gambia found the positive effects of full house screening and screened ceilings, including open eaves, on anemia but not on parasitemia [15]. The parasitemia result might be partially due to use of non-insecticidal nettings for screening. Further, the Gambia study randomized households rather than communities or villages, which might exclude the community effects [45]. Although the differences in parasitemia between the treatment arms were not statistically significant in the Gambia study, the parasitemia of the whole study area (including both intervention and control households) was much reduced in the second year, suggesting the appearance of community effects. To ensure community effects, we modified the boundaries and established 300-m buffer zones along them to minimize the spillover effect between clusters (Fig. 1) [44, 45]. We will install ceiling nets in all houses in the intervention clusters including houses without eligible children except houses without open eaves. Further, we will use the netting material incorporated with insecticide. Accordingly, for the sample size calculation, we aimed for 50% reduction in both entomological and epidemiological endpoints in the treatment arms. The impact of the reduction is considerable for malaria control, and it is achievable when referring to the results of the past studies [13–15, 28, 29].

In contrast, the community-level randomization limited the number of target clusters to 4 for each treatment arm and control arm. Concerning the spatial heterogeneities of infected children and vector community, each cluster could have been divided to a few smaller clusters, which may reduce the imbalances between clusters and the sample size of each cluster. This may also allow a parametric analysis. However, we avoided the risk of introducing an uncomfortable atmosphere and feelings among the residents within the same community through dividing them into multiple treatment groups. Despite the buffer zone, a smaller cluster may increase the risk of spillover from the adjacent clusters. Therefore, adjusting the imbalances using the baseline data is essential to increase the statistical power.

We planned to conduct the epidemiological baseline study after the short rainy season. As shown in the present preliminary study, we expected the high numbers of vectors and infected children during the period. Nevertheless, higher transmission risk is usually associated with the long rainy season. To evaluate the impact of the interventions more clearly, we planned the first post-intervention epidemiological study immediately after the long rainy season.

## Conclusion

Based on the results from the present preliminary study, we concluded that the study design of the cRCT is feasible. As the number of clusters is limited, we will apply a two-stage procedure with the baseline data to evaluate each intervention.

## Data Availability

Not applicable

## Abbreviations

95% CI: 95% credible interval
*An.*: *Anopheles*
cRCT: Cluster randomized controlled trial
IRS: Indoor residual spraying
ITN: Insecticide-treated net
kdr: Knockdown resistance
LLIN: Long-lasting insecticidal net
*P.*: *Plasmodium*
PBO: Piperonyl butoxide
PCR: Polymerase chain reaction
PCRpfPR: *Plasmodium falciparum* PCR-positive prevalence
RDT: Rapid diagnostic test
RDTpfPR: *Plasmodium falciparum* RDT-positive prevalence
sd: Standard deviation
*s. l.*: *Sensu lato*
*s. s.*: *Sensu stricto*
WHO: World Health Organization
